# A low-endotoxic *Salmonella* vector with dual bacterial-host promoter expression of *Lawsonia intracellularis* antigens elicits protective immunity in a murine model

**DOI:** 10.1186/s13567-026-01726-w

**Published:** 2026-03-21

**Authors:** Muhammad Bakhsh, Amal Senevirathne, John Hwa Lee

**Affiliations:** College of Veterinary Medicine, Jeonbuk National University, Iksan, 54596 Republic of Korea

**Keywords:** *Lawsonia intracellularis*, proliferative enteropathy, *Salmonella*, antigen delivery, dual promoter, immunogenicity, protection

## Abstract

*Lawsonia intracellularis*, the etiological agent for proliferative enteropathy, remains a major cause of productivity loss and economic burden in the global swine industry. Currently available vaccines provide suboptimal protection and raise safety concerns, highlighting the need for improved immunization strategies. In this study, we developed *Salmonella* Typhimurium-based vaccine candidates against *L. intracellularis* in a murine model, incorporating immunoprotective epitopes from the surface autotransporter protein LatA, the flagellar protein FliC, and the heat shock protein 60 (HSP60). These antigens were expressed using dual bacterial-host plasmids, pJHL270 and pJHL305, containing prokaryotic (Ptrc) and eukaryotic (CMV) promoters to ensure both extracellular and intracellular antigen presentation. The attenuated *Salmonella* delivery strain JOL3086 (Δ*lon* Δ*pagL*) exhibited a high safety profile with markedly reduced endotoxicity. Western blot analysis confirmed antigen expression in both bacterial and mammalian systems in vitro. Immunization with the recombinant *Salmonella* strains elicited significant humoral (systemic IgG and mucosal IgA) and cell-mediated (CD4^+^, CD8^+^ T cells) immune responses, accompanied by significantly increased levels of *Ifng*, *Il6*, *Il4*, and *Il10* cytokines. Among the three vaccine strains, JOL3149 (pJHL305 expressing FliC-HSP60) induced the most pronounced immunological responses and conferred superior protection in C57BL/6 mice challenged with *L. intracellularis*, as evidenced by decreased fecal bacterial shedding, reduced intestinal bacterial load, and alleviated intestinal lesions postchallenge. Collectively, these findings demonstrate the potential of the *Salmonella*-mediated dual expression platform as a safe and effective delivery system for *L. intracellularis* antigens, offering a promising foundation for next-generation vaccines against *L. intracellularis*.

**Supplementary Information:**

The online version contains supplementary material available at 10.1186/s13567-026-01726-w.

## Introduction

*Lawsonia intracellularis*, an obligate intracellular pathogen, invades the small intestine of pigs [1], horses [2], hamsters [3], rabbits [4], and chickens [5]. In pigs, the bacterium causes porcine proliferative enteropathy (PPE), resulting in significant production losses for the global swine industry [6, 7]. Although the disease was first identified in pigs in 1931, Alan Rowland and Gordon Lawson described the true nature of PPE in 1973, when they observed curved-shaped intracellular bacteria [8]. There are three clinical forms of PPE: porcine intestinal adenomatosis (PIA), proliferative hemorrhagic enteropathy (PHE), and necrotic enteritis (NE) [9–12]. *L. intracellularis* primarily colonizes the enterocytes in the distal ileum and, occasionally, in the jejunum, cecum, and colon, leading to increased cellular proliferation of immature crypt cells [13]. The infection manifests as diarrhea, initially watery and progressing to bloody, as well as stunted growth and, in some cases, mortality in young pigs [14]. A significantly higher number of incidents have been reported in intensive pig farms, while the majority of infections remain subclinical [15]. The herd-level prevalence remained around 48%, with occasional values reaching 100% [16, 17]. It is generally neglected by researchers owing to its low mortality rate and difficulties in culturing under laboratory conditions, resulting in limited progress in developing effective preventive strategies and therapeutic interventions [18].

Owing to these circumstances, vaccination is one of the most plausible ways of mitigating a disease such as PPE in vulnerable animal populations [19]. Currently, live attenuated vaccine (Enterisol^®^ Ileitis, Boehringer Ingelheim) and inactivated vaccine (Porcilis^®^ Ileitis, Merck Animal Health) are available for pigs’ vaccination against the disease. The live attenuated vaccine has been available since 2001 and provides a high level of protection. However, it has some negative implications, including bacterial shedding that can lead to environmental contamination and the further spread of the disease among immunocompromised individuals [20]. In addition, the inactivated *L. intracellularis* vaccine (Porcilis^®^ Ileitis) has been reported to induce less protective immunity than the live vaccine [14, 21–23]. These conditions justify the development of novel immunization strategies to improve both effectiveness and safety, thereby enhancing animal productivity.

Despite the challenges associated with *L. intracellularis* cultivation for antigen isolation and evaluation, the availability of modern sequencing data and in silico immunological tools facilitated the selection of potential antigen candidates during vaccine development. Most of *L. intracellularis* antigens are less well characterized as vaccine candidates; therefore, thorough evaluation of their immunogenicity and suitability is essential in new vaccine development. Previous studies have reported that convalescent serum collected from pigs contained neutralizing antibodies against the *Lawsonia* autotransporter A (LatA) protein [24], which reflects the antigenicity of this protein. It is a bacterial surface protein with a molecular weight of 72 kDa [24–26]. As a strategy to promote antigen processing, we selected several immunodominant epitopes based on B and T cell epitope scanning approaches using swine immune parameters, rather than the complete LatA protein [27]. The bacterium also possesses FliC, a flagellin-associated protein that interacts with host TLR-5 receptors during immune activation [28]. It possesses an extracellular unipolar flagellum that facilitates host-cell invasion and broader infection of its natural hosts [29, 30]. This triggers the secretion of proinflammatory cytokines, subsequently promoting the activation of T cell-mediated adaptive immune responses [31, 32]. To ensure full TLR-5 activation, the complete FliC protein was chosen for the current vaccine study. In our previous mass spectrometry investigation, we identified HSP60/GroEL as another immunodominant protein with the potential to induce protective immune responses against *L. intracellularis* infection [33]. The localization of HSP60 can vary in response to environmental stimuli, appearing in the cytosol, on the cell surface, or in the secretome [34]. Here, too, the complete HSP60 protein was used as an immunogen to preserve structural integrity and promote a natural-like immune response upon immunization. According to previous studies, HSPs have demonstrated the ability to elicit protective immunity against a wide range of pathogens [32, 35–38], justifying their selection as protective antigens for this study. Overall, three promising antigens were selected for the current vaccine development project: LatA epitopes, full FliC, and full HSP60.

Vaccine development is undergoing a paradigm shift, with live attenuated vectors, especially those derived from *Salmonella*, recognized for their ability to induce strong immunoprotective responses against numerous pathogens [39, 40]. For a successful immune response, the selected antigens must be efficiently delivered to antigen-presenting cells. For this purpose, we developed an attenuated *Salmonella* Typhimurium strain containing deletions in *lon, pagL,*, and *asd* using the lambda red recombination technique. Herein, both *lon* and *pagL* are attenuation markers, while *asd* is the antibiotic-free selection marker. *Salmonella* has an intrinsic propensity to invade professional antigen-presenting cells (APCs) such as dendritic cells and macrophages [41]. Hence, heterologous antigens can be secreted directly into APCs for immune elicitation. The Lon protease is a global regulator essential for the normal physiology of *Salmonella*, and its deletion leads to overexpression of certain virulence factors, which can be a favorable feature for efficient invasion. However, the strain cannot resist chronic infection due to its lack of mitigation towards oxidative stress conditions [42–44]. Furthermore, the *pagL* gene encodes a lipid A deacylase enzyme. It modifies the lipid A structure, a known TLR4 agonist, thereby causing significantly lower endotoxic responses [45]. The deletion of the *asd* gene enables antibiotic-free selection procedures for the subsequent delivery and expression of desired antigens [46, 47]. Deleting these genes not only attenuates *Salmonella* virulence but also makes them promising candidates for safe vaccine antigen delivery. We confirmed successful elicitation of antigen response and ensured that these vectors are effective against *L. intracellularis* infection. In this way, they enhance the promising application of *Salmonella*-based vaccines in veterinary medicine and, potentially, in human medicine [40, 48, 49].

The current challenge study was conducted in the C57BL/6 mouse model, as they are susceptible to *L. intracellularis* infection [33], although the infection is not as severe as in pigs. Challenge material was collected from *L. intracellularis*-infected pig tissues to test the vaccine candidates against a natural wild-type strain. In this study, we developed both the vaccine antigens and the *Salmonella* delivery strain and demonstrated that this vaccination modality is applicable to practical farming conditions, as it can reduce the disease severity of both *Salmonella* and *L. intracellularis* in the same enteric environment. Further development can focus on dual protection under field conditions and on evaluating the vaccine’s efficacy in real-world settings.

## Materials and methods

### Development of a therapeutic ST delivery system by lambda red recombineering

All bacteria were cultured in Luria–Bertani (LB) medium (BD, Sparks, USA) at 37 °C in a shaking incubator. The bacterial strains used have been listed in Table 1. The bioengineered ST strain JOL3090 (ST: ∆*lon*∆*pagL*∆*asd*) used in this study was developed by deleting the *pagL* and *asd* genes from the *lon-*deleted parent strain JOL909 using the lambda red recombinant technology. Briefly, the *pagL* gene was replaced by a Frt-flanked chloramphenicol resistance cat^R^ using pKD46 containing recombinase-competent strains. The template for Frt-Cat-Frt was obtained using the pKD3 plasmid and electroporated into *Salmonella* using stringent conditions (2.2 kV, 0.4 ms, 200 Ohms, 25 µF, 1 mm gap cuvettes). The gene deletion was confirmed using the specific inner primers (Table 2). Ultimately, the *catR* gene was evicted using the pCP20 plasmid, as described by Datsenko et al. [50]. A similar method was used to delete the *asd* gene to generate an auxotrophic *Salmonella* strain, which was finally named JOL3090. The growth of auxotrophs was sustained by adding diaminopimelic acid (DAP; Sigma-Aldrich, St. Louis, MO, USA) at 50 µg/mL.

**Table 1:** **List of bacterial strains and plasmids used in this study**

Strains/plasmids	Genotype characteristics	References
*S*. Typhimurium		
JOL401 JOL909	Wild-type *S. Typhimurium* *∆lon* mutant of *S.* Typhimurium	Lab stock Lab stock
JOL3086	*∆lon, ∆pagL* mutant of *S. Typhimurium*	This study
JOL3090	*∆lon, ∆pagL*,* ∆asd* mutant of *S. Typhimurium*	This study
JOL3111	JOL3090 containing pJHL270 as vector control	This study
JOL3112	JOL3090 containing pJHL270—Pro LatA epitopes + Eu HSP60, and expressing LatA epitopes and HSP60	This study
JOL3113	JOL3090 containing pJHL270—Pro FliC + Eu HSP60, and expressing FliC and HSP60	This study
JOL3148	JOL3090 containing pJHL305 as vector control	This study
JOL3149	JOL3090 containing pJHL305—Pro FliC + Eu HSP60, and expressing FliC and HSP60	This study
*E. coli*		
BL21(DE3) pLysS	F^–^, *omp*T, *hsd*S_B_ (r_B_^–^, m_B_^–^), *dcm, gal*, λ (DE3), pLysS, Cm^r^	Promega, USA
*E. coli* 232	F − λ − φ80 Δ(lacZYA-argF) endA1 recA1 hadR17 deoR thi-1 glnV44 gyrA96 relA1 ΔasdA4	Lab stock
DH5α	*E. coli* F^−^Φ80dlacZΔM15Δ (lacZYA-argF) U169 recA1 endA1 hsdR17(rk − , mk^+^) phoA supE44 thi1 gyr A96 relA1λ −	Lab stock
JOL2723	DH5α carrying pET28a-HSP60	This study
JOL3101	DH5α carrying pET28a-LatA epitopes	This study
JOL3102	DH5α carrying pET28a-FliC	This study
JOL2724	DE3 carrying pET28a-HSP60	This study
JOL3105	DE3 carrying pET28a-LatA epitopes	This study
JOL3106	DE3 carrying pET28a-FliC	This study
JOL3099	*E. coli* 232 carrying pJHL270—Pro LatA epitopes + Eu HSP60	This study
JOL3100	*E. coli* 232 carrying pJHL305—Pro FliC + Eu HSP60	This study
JOL3145	*E. coli* 232 carrying pJHL270—Pro FliC + Eu HSP60	This study
Plasmids		
pKD46	Ori101-repA101ts; encodes Lambda red genes (exo, bet, gam); native terminator (tL3); arabinose-inducible for expression (ParaB); bla	Lab stock
pKD3	oriR6K gamma, bla (ampR), rgnB (Ter), catR, FRT	Lab stock
pCP20	Helper plasmid, contains a temperature-inducible flp gene for removing the FRT flanked chloramphenicol gene	Lab stock
pET28a ( +)	IPTG-inducible expression vector; Kanamycin resistant	Novagen, USA
pJHL270	asd + , CMV eukaryotic promoter, Ptrc prokaryotic promoter, BlaSS secretory signal sequence, pBR322 ori	Lab stock
pJHL305	asd + , CMV promoter, RdRp complex (nsp1–4) SV40 promoter, 26Spromoter, pBR322 ori	Lab stock

**Table 2:** **List of primers used in this study**

Gene	Primers	Reference
*pagL* inner	Forward: CAGATCTCTTTTGCTGCGGG Reverse: AAAAGCCCCAAAGTTCCAGC	[75]
*pagL* outer	Forward: TGGATGTGCCTGAACAACACT Reverse: TTAGCCTCCCTGTCGCCATA	[75]
*asd* inner	Forward: CATGGTAGAGGAGCGCGATT Reverse: TACCGCCCACAAAGGTCTTC	[75]
*asd* outer	Forward: GCGACGGAAATGATTCCCTT Reverse: AAGCTACCCTTAAAGAATAGCC	[75]
*Lawsonia aspA*	Forward: GCTGTGGATTGGGAGAAATC Reverse: CAAGTTGACCAGCCTCTGC	[76]
*Ifn*	Forward: AGACAATGAACGCTACACAC Reverse: TCTTTTCTTCCACATCTATGCC	[44]
*Tnf*	Forward: CATCTTCTCAAAATTCGAGTGACAA Reverse: TGGGAGTAGACAAGGTACAACCC	[77]
*Il1b*	Forward: TTCACCATGGAATCCGTGTC Reverse: GTCTTGGCCGAGGACTAAGG	[78]
*Il4*	Forward: ACGGATGCGACAAAAATCAC Reverse: ACCTTGGAAGCCCTACAGAC	[44]
*Il6*	Forward: CAGAATTGCCATTGCACAACTCTTTTCTCA Reverse: AAGTGCATCATCGTTGTTCATACA	Lab stock
*Il10*	Forward: GGTTGCCAAGCCTTATCGGA Reverse: ACCTGCTCCACTGCCTTGCT	[48]
*β*-actin	Forward: AGAGGGAAATCGTGCGTGAC Reverse: CAATAGTGATGACCTGGCCGT	[77]

### Vaccine design and in silico analysis of *L. intracellularis* antigens

The gene sequences of LatA (WP_011526732.1), FliC (WP_263615834), and HSP60 (AB218756) were obtained from the National Library of Medicine, National Center for Biotechnology Information. Molecular simulations of LatA, FliC, and HSP60 proteins with toll-like receptor 4 (TLR-4) and TLR-5 were analyzed using the ZDOCK server and Discovery Studio. Earlier studies have assessed the LatA and FliC antigens and demonstrated their immunoprotective effects [21, 25, 26, 31, 51]. This time, we selected immunodominant LatA epitopes, as well as the complete FliC sequence, and cloned them into two different vaccine constructs, both of which were cloned into the prokaryotic region of the plasmids. Briefly, six immunodominant epitopes were selected on the basis of B- and T-cell epitope predictions from the Immune Epitope Database (IEDB [21] using default parameters, with pig immune parameters. Linear B-cell epitopes were predicted using the BepiPred 2.0 algorithm, and peptides with scores > 0.5 were selected. In contrast, T-cell epitopes were identified using the IEDB MHC class I and II prediction methods, selecting peptides with favorable percentile ranks and predicted binding affinities. The selected immunodominant epitopes spanning amino acid residues 45–83, 163–186, 222–242, 684–712, 734–749, and 784–808 were connected via glycine linkers to form a single chimeric amino acid construct. Previously, we detected HSP60/GroEL by mass spectrometry as an immunodominant protein in *L. intracellularis* lysate [33]*.* Therefore, HSP60 was used in both constructs for the eukaryotic expression. The sequences were codon-optimized and custom-synthesized by Cosmogenetech, Republic of Korea.

The overall structural integrity and fusion product of the selected antigens were predicted in silico using the bioinformatics tools. The BepiPred Linear Epitope Prediction tool was used to predict linear B-cell epitopes of the antigens, which identifies antigenic regions on the basis of amino acid propensity score [27]. The SwissModel server was used to evaluate the structural stability of the antigens, employing homology-based modeling to assess folding feasibility [52]. The structures were then validated to identify energetically favorable regions using Ramachandran plots via the Procheck server at EMBL-EBI [53]. The ProSA-web server was used to evaluate the overall quality of the predicted protein structures [54]. Furthermore, the solubility and antigenicity of the constructs were verified using Protein-Sol and Vaxigen 2.0, respectively [55, 56].

### *L. intracellularis* antigens delivery strategy using dual bacterial-host expression systems

Dual bacterial-host promoter expression vector plasmids, pJHL270 and pJHL305, which contain cytomegalovirus (CMV) and Ptrc promoters for eukaryotic and prokaryotic expression, respectively, were used to express *L. intracellularis* antigens. In both plasmids, the Ptrc promoter produces antigen expression from *Salmonella,* and the CMV promoter is deployed for antigen expression from the eukaryotic counterpart. Moreover, pJHL305 harbors the RNA-dependent RNA polymerase (RdRp) of Semliki Forest Virus, facilitating better mRNA amplification. This approach promotes antigen presentation, facilitating intracellular and extracellular antigen presentation to elicit a Th1/Th2-mediated immune response.

LatA epitopes, FliC, and HSP60 were cloned into the suitable MCS regions of the dual-expression plasmids, pJHL270 and pJHL305. Two vaccine constructs were designed using the pJHL270. The first construct (pJHL270: Pro LatA + Eu HSP60) was developed by cloning LatA epitopes for prokaryotic and HSP60 for eukaryotic expression. The second construct (pJHL270: Pro FliC + Eu HSP60) was designed by cloning FliC for prokaryotic and the HSP60 for eukaryotic expression, respectively. The same cloning strategy as the second construct was applied to develop a third one in the pJHL305 plasmid (pJHL305: Pro FliC + Eu HSP60). The resulting clones were transformed into *E. coli* strain lacking *asd,* and were electroporated into the attenuated ST strain at 1800 kV (BTX, Harvard Apparatus, USA). The respective empty vectors pJHL270 and pJHL305 were electroporated to obtain vector control strains.

### Protein purification and preparation of polyclonal antibodies

The antigenic proteins were expressed in *E. coli* using the pET28a (+) vector system (Novagen, USA) according to the standard procedure. Briefly, the target genes were cloned into pET28a (+), and the *E. coli* DH5-α strain was transformed with the cloned plasmid. The plasmid was then extracted, PCR-confirmed, and transformed into *E. coli* BL21 (DE3 cells). Bacterial cultures were induced to express the proteins by the addition of isopropyl β-D-1-thiogalactopyranoside (IPTG) to a final concentration of 1 mM, followed by incubation for 5 h at 37 °C. Protein expression was confirmed by sodium dodecyl sulfate–polyacrylamide gel electrophoresis (SDS-PAGE).

Ni–NTA affinity chromatography (Bio-Rad, Hercules, CA, USA) was used for protein purification, and the concentrations were determined using the Bradford assay [57]. SPF New Zealand white rabbits, aged 2 months, were used to prepare polyclonal antibodies against each protein. Briefly, purified antigens (250 μg) were emulsified in Complete Freund’s Adjuvant (CFA) and were injected into the rabbits subcutaneously. At 2 weeks after the primary injection, booster doses were administered using Incomplete Freund’s Adjuvant (IFA) via the same route. Hyperimmune sera, as polyclonal antibodies, were collected 2 weeks after the booster dose for subsequent experiments.

### In vitro expression of vaccine constructs

Bacterial cell lysates from vaccine constructs and their vector controls were used to validate prokaryotic antigen expression. Briefly, the bacterial strains were cultured to the logarithmic growth phase and harvested by centrifugation at 2500 × *g* for 15 min. The cell pellets were washed with phosphate-buffered saline (PBS) and sonicated for 30 s with 10-s intervals at 40% amplitude. The resulting lysates were collected by centrifugation and further analyzed by western blotting.

Similarly, RAW 264.7 cells were infected with the vaccine strains at 40 MOI to confirm eukaryotic antigen expression. After 4 h of infection, the cells were treated with gentamicin to kill the extracellular bacteria. Furthermore, the cells were incubated at 37 °C with 5% CO_2_ for 48 h. The protein was extracted using PRO-PREP™ Protein Extraction Solution (iNtRON Biotechnology) according to the manufacturer’s instructions, and the expression was detected by western blotting.

For western blotting, proteins were mixed with 5 × SDS buffer, boiled at 96 °C for 5 min, and subjected to 12% SDS-PAGE. The separated proteins were transferred to a polyvinylidene difluoride (PVDF) membrane and blocked with 5% skim milk for 1 h at room temperature. The membranes were incubated overnight at 4 °C with hyperimmune rabbit sera against each protein (1:500), followed by HRP-conjugated anti-rabbit IgG antibodies (1:6000) (Invitrogen, Massachusetts, USA). The expected protein size was detected by adding western blotting substrate, WESTSAVE Gold (Abfrontier Co., Ltd., Korea), and chemiluminescent exposure using ChemiDoc Imaging (Cytiva, Marlborough, MA, USA).

### Animals and ethics statement

The 5-week-old, specific-pathogen-free (SPF), C57BL/6 female mice were obtained from Koatech Laboratory Animals, Inc. (Korea) and maintained at the Animal Housing Facility, College of Veterinary Medicine, Jeonbuk National University, South Korea. The study was approved by the Jeonbuk National University Animal Ethics Committee (NON2022-024-002). The mice were kept in a 12 h light–dark cycle and provided a standard chow diet and potable water ad libitum.

### Adhesion and invasion assays

The adhesion and invasion abilities of the *Salmonella* delivery strain were evaluated using human epithelial (HEp-2) and porcine kidney (PK-15) cell lines. Briefly, the cells were cultured in a 24-well plate. Overnight broth cultures of the bacterial strains were freshly grown to mid-log phase in LB broth and then used to infect cells at a 40 MOI. The cells were infected for 30 min, then washed with PBS before the monolayers were lysed with 0.1% Triton X-100 for 10 min. Adherent bacteria were counted by plating onto LB agar.

The invasion assay was performed as described elsewhere [58] with some modifications. The cells were infected as described above, then incubated for 2 h, followed by 2 h of gentamicin (Sigma, St. Louis, MO, United States) treatment to kill extracellular bacteria. The cells were then lysed with 0.1% Triton X-100 for 10 min. The number of invaded cells was quantified by serial dilution and plating on LB agar.

### Assessment of virulence and endotoxicity of the ST delivery system

The virulence of the mutant *Salmonella* delivery strain was assessed in 6-week-old C57BL/6 female mice. The mice were divided into three groups (*N* = 24, *n* = 8) and orally inoculated with 10^7^, 10^8^, or 10^9^ CFU of the mutant strain (JOL3086) in a volume of 100 μL PBS. Mice were monitored daily, and the corresponding mortality was recorded for 14 days. Humane end points were applied to minimize suffering.

To determine safety, bacterial colonization, and endotoxicity, 6-week-old C57BL/6 mice were randomly divided into three groups (*N* = 24, *n* = 8). The mice were orally inoculated with 1 × 10^7^ CFU/100 μL, either with JOL401 or JOL3086. A group of uninoculated mice served as the naive control. Following the injection, animals were monitored daily for survival until the end of the experiment. On days 3 and 5 postinoculation, spleen and liver samples were weighed and homogenized in 1 mL of buffered peptone water, and 100 µL was serially diluted and plated on BGA plates to count colony-forming units (CFUs). A portion of the spleen was used to isolate RNA to assess endotoxicity by determining the proinflammatory cytokines *Tnf* and *Il1β* using quantitative real-time PCR (qRT-PCR) with the primers listed in Table 2. Spleen and liver samples were stored in 10% neutral buffered formalin, processed, and stained with hematoxylin and eosin (H&E) for histopathological analysis.

### H_2_DCFDA assay for intracellular ROS detection

Intracellular reactive oxygen species (ROS) were evaluated using 2′,7′-dichlorodihydrofluorescein diacetate (H_2_DCFDA) (Thermo Fisher Scientific, USA). Briefly, PK-15 cells were cultured in 6-well plates and infected with JOL401 and JOL3086 at 40 MOI. After incubation for 2 h, cells were washed with PBS to remove extracellular bacteria and debris.

A 10 mM H_2_DCFDA stock solution was prepared in anhydrous DMSO and stored at −20 °C in the dark. Immediately before use, the stock was diluted to a final concentration of 10 µM in serum-free, phenol red-free Dulbecco’s modified eagle medium (DMEM). Cells were incubated with the working solution for 30 min at 37 °C in the dark. After incubation, the cells were washed with PBS and examined under a fluorescence microscope. Images were acquired in bright-field, fluorescence, and merged modes using identical exposure parameters.

### Assessment of *Salmonella*-induced cytotoxicity

*Salmonella*-mediated cytotoxic effects were evaluated using the IncuCyte live-cell imaging system (Essen Bioscience, USA). MARC-145 cells were seeded in 24-well culture plates at a density of 5 × 10^4^ cells. Cells were infected with wild-type (WT) *Salmonella* (JOL401) and delivery mutant (JOL3086) at a multiplicity of infection (MOI) of 40 for 2 h, followed by two washes with PBS. Extracellular bacteria were eliminated by treatment with gentamicin (100 µg/mL) for 2 h. Subsequently, propidium iodide (5 µL/mL; BD Biosciences, CA, USA) was added to the cultures, and cytotoxicity was monitored by live-cell imaging at 4 h intervals for a total duration of 24 h.

### Animal experiments

The 6-week-old SPF C57BL/6 female mice were used to screen and evaluate immunoprotective *Salmonella*-based vaccine strains expressing *L. intracellularis* antigens. In the initial screening phase, mice were randomly assigned to five groups (*N* = 60, *n* = 12 per group) and immunized orally with JOL3111, JOL3112, JOL3113, or PBS, with an unimmunized group serving as a naive control. Each mouse received 1 × 10^7^ CFU of the respective vaccine strain formulated in 100 µL PBS, administered twice at 2-week intervals. In the third week of the booster dose, mice in each group were challenged orally with 5 × 10^7^ *L. intracellularis*, as determined by qRT-PCR. The mice were monitored for clinical signs, antibody response, and fecal bacterial shedding to identify a highly immunoprotective strain.

In the subsequent evaluation phase, the highly immunoprotective *L. intracellularis* antigens identified earlier were incorporated into the pJHL305 plasmid. Mice were divided into seven groups (*N *= 84, *n* = 12) and immunized orally with JOL3148, JOL3149, JOL3111, JOL3112, JOL3113, PBS, or left naive, following the same immunization and challenge procedures described above to assess the comparative protective efficacy of the vaccine strains.

### Enzyme-linked immunosorbent assay (ELISA)

At 14- and 28-day postinoculation, blood was collected from the mice’s retro-orbital sinus by trained personnel and allowed to clot at room temperature for 1 h. The sera were collected and stored at −80 °C, after centrifugation at 2500 × *g* for 10 min at 4 °C. Furthermore, vaginal wash samples were collected for measuring secretory IgA. An indirect ELISA was performed to assess *L. intracellularis* antigen-specific systemic immunoglobulin G (IgG) and mucosal immunoglobulin A (IgA) antibody responses in mice sera and vaginal wash samples, respectively. LatA, FliC, and HSP60 purified proteins were coated (500 ng/well) in 96-well plates at 4 °C overnight in a carbonate-bicarbonate buffer at pH 9.6. The plates were blocked with 5% skim milk for 1 h at room temperature, followed by three washes with PBS containing 0.05% Tween 20 (PBST). Appropriate dilution of immunized sera and vaginal wash samples was added and incubated at 4 °C overnight. The wells were treated with HRP-conjugated goat anti-mouse antibodies (Southern Biotech, Alabama, USA) at a 1:4000 dilution and incubated at 37 °C for 1 h, after three washes with PBST. The color was developed through enzymatic reactions with O-phenylenediamine (Sigma-Aldrich, Missouri, USA) and stopped with 50 μL of 2N sulfuric acid. An automatic ELISA spectrophotometer (Tecan) was used to measure absorbance at 492 nm.

### Fluorescence-activated cell sorting (FACS) analysis for CD4^+^ and CD8^+^ T cells

At 2 weeks after booster immunization, splenocytes from four mice per group were analyzed by FACS to determine CD4^+^ and CD8^+^ T cell populations. The harvested splenocytes (1 × 10^5^/ well) were cultured in a 96-well plate in Roswell Park Memorial Institute (RPMI) media containing 10% FBS. The cells were stimulated with the purified proteins (500 ng/well) and incubated at 37 °C with 5% CO_2_ for 24 h. The cells were then washed and stained with CD3e-PE, CD4-PerCPVio700, and CD8a-FITC antibodies (Miltenyi-Biotec, Germany) at 4 °C for 30 min in the dark. The CD3^+^ T cell population was gated to analyze the CD3^+^CD4^+^ and CD3^+^CD8^+^ T cell subpopulations accordingly.

### Quantitative real-time PCR (qRT-PCR) analysis for cytokine expression

Harvested splenocytes were cultured in 12-well plates (1 × 10^6^/ well) and stimulated with purified proteins (500 ng/well) and incubated at 37 °C with 5% CO_2_ for 24 h. Total RNA was extracted using Hybrid-R^TM^ (GeneAll, South Korea), following the manufacturer’s instructions, and complementary DNA was synthesized using a reverse transcription master premix (Elpis Biotech, Korea). Quantitative real-time PCR (qRT-PCR) was performed to assess mRNA levels of *Ifng, Il6, Il4,* and *Il10* using SYBR Green Master Mix (ELPIS Biotech, South Korea), using the respective primers listed in Table 2.

### MTT assay

The 3-(4, 5-dimethylthiazol-2-yl)-2, 5-diphenyl tetrazolium bromide (MTT, Sigma-Aldrich) assay was performed to measure splenocyte proliferative responses at 2 weeks after booster immunization. Harvested splenocytes were stimulated with LatA, FliC, and HSP60 purified proteins and incubated for 72 h at 37 °C with 5% CO_2_. The cells were treated with MTT reagent (5 mg/mL) and further incubated for 4 h. DMSO (100 μL) was added to each well to dissolve formazan, and colorimetric measurements were noted using a microplate reader at 570 nm wavelength. The stimulation index was calculated by dividing the values from stimulated cells by those of unstimulated cells.

### Challenge study

The challenge material was derived from the intestine of pigs infected with *L. intracellularis*, as confirmed by PCR and immunohistochemistry (IHC). Briefly, the affected portions of the intestine showing clinical indications were scraped and diluted in Dulbecco’s modified Eagle medium (DMEM) (Lonza, North Carolina, USA). The mucosa was then homogenized with a blender and frozen at −80 °C for further studies. Moreover, a preliminary experiment was conducted to measure the estimated dose for challenge studies in mice. Hence, the homogenate containing approximately 5 × 10^7^ *L. intracellularis* was inoculated orally to each mouse, as assessed by qRT-PCR.

### Evaluation of *L. intracellularis* load in challenge mice

To evaluate *L. intracellularis* shedding, fecal samples were collected on days 7, 14, and 21 postchallenge, and the bacterial load was determined by qRT-PCR, targeting the aspartate ammonia-lyase (*aspA*) gene using the primers listed in Table 2. Briefly, genomic DNA was extracted from fecal samples using the Exgene Stool DNA Mini Kit (GeneAll Biotechnology, South Korea) according to the manufacturer’s instructions. The *aspA* gene was amplified by qRT-PCR using SYBR Green Master Mix (ELPIS Biotech, South Korea), with cycling conditions as follows: 5 min at 95 °C, followed by 40 repeats of 95 °C for 10 s, 60 °C for 30 s, 72 °C for 30 s, and a final 72 °C for 5 min. A standard curve was generated for the quantification of gDNA per gram of feces.

Moreover, the bacterial load in ileal tissue was measured on day 21 postchallenge. Briefly, genomic DNA was extracted using the AccuPrep Genomic DNA Extraction Kit (Bioneer, South Korea), and qRT-PCR was performed to measure the bacterial load in the ileum as described earlier.

### Hematoxylin and eosin (H&E) staining

Histopathological examination of the intestinal tissues was performed to assess the potential damage caused by *L. intracellularis*. On day 21 postchallenge, intestinal samples were collected, gently flushed with phosphate-buffered saline (PBS), and fixed in 10% neutral-buffered formalin for 24 h at room temperature. Fixed tissues were processed through a graded ethanol series, cleared in xylene, and embedded in paraffin wax according to standard histological procedures. Paraffin-embedded tissues were sectioned at a thickness of 4 μm using a microtome and mounted on glass slides. Tissue sections were deparaffinized, rehydrated, and stained with hematoxylin and eosin (H&E). Stained sections were examined under a light microscope to provide detailed insights into structural and pathological changes.

### Comparative protection study using a commercial *L. intracellularis* vaccine

To assess the relative protective efficacy of the lead vaccine candidate, an additional protection experiment was conducted using a commercial live *L. intracellularis* vaccine (Enterisol^®^ Ileitis, Boehringer Ingelheim). The 6-week-old SPF C57BL/6 female mice were randomly divided into four groups (*N* = 32, *n* = 8 per group): JOL3149-immunized, commercial vaccine (CV)-immunized, PBS, and naive control. In the CV group, mice were orally treated with 200 μL attenuated commercial vaccine (10^4.9^ TCID_50_/dose) according to the manufacturer’s instructions. The operation in the other groups and the challenge protocol were performed as described above. Protective efficacy was evaluated on the basis of fecal bacterial shedding and intestinal histopathological lesions following challenge.

### Statistical analysis

Statistical analyses were conducted using GraphPad Prism v8.0 (GraphPad Software, CA, USA). For comparisons among multiple groups, one-way analysis of variance (ANOVA) followed by Tukey’s post hoc test was used. For time-course data, two-way ANOVA followed by Sidak’s or Dunnett’s multiple-comparison test, as appropriate, was used to compare differences among groups. The data are presented as mean ± standard deviation (SD), and *p*-values < 0.05 were considered significant. Sample sizes (*n*) for each experiment are indicated in the figure legends.

## Results

### Engineering of ST delivery strain

The bacterial strains and plasmids used in this study are listed in Table 1. The ST train was genetically attenuated by introducing defects in LPS structure using the highly efficient lambda red recombination approach [50]. The *pagL* gene is expected to inhibit the deacylation of lipid A, thus potentially preventing LPS from undergoing key structural modifications and altering its biological functions. The parent strain JOL909 was used to delete the *pagL* and *asd* genes via recombineering, and the gene deletions were confirmed by PCR (Additional file 1) using specific outer primers listed in Table 2.

### In silico analysis of *L. intracellularis* antigens

**Figure 1:**
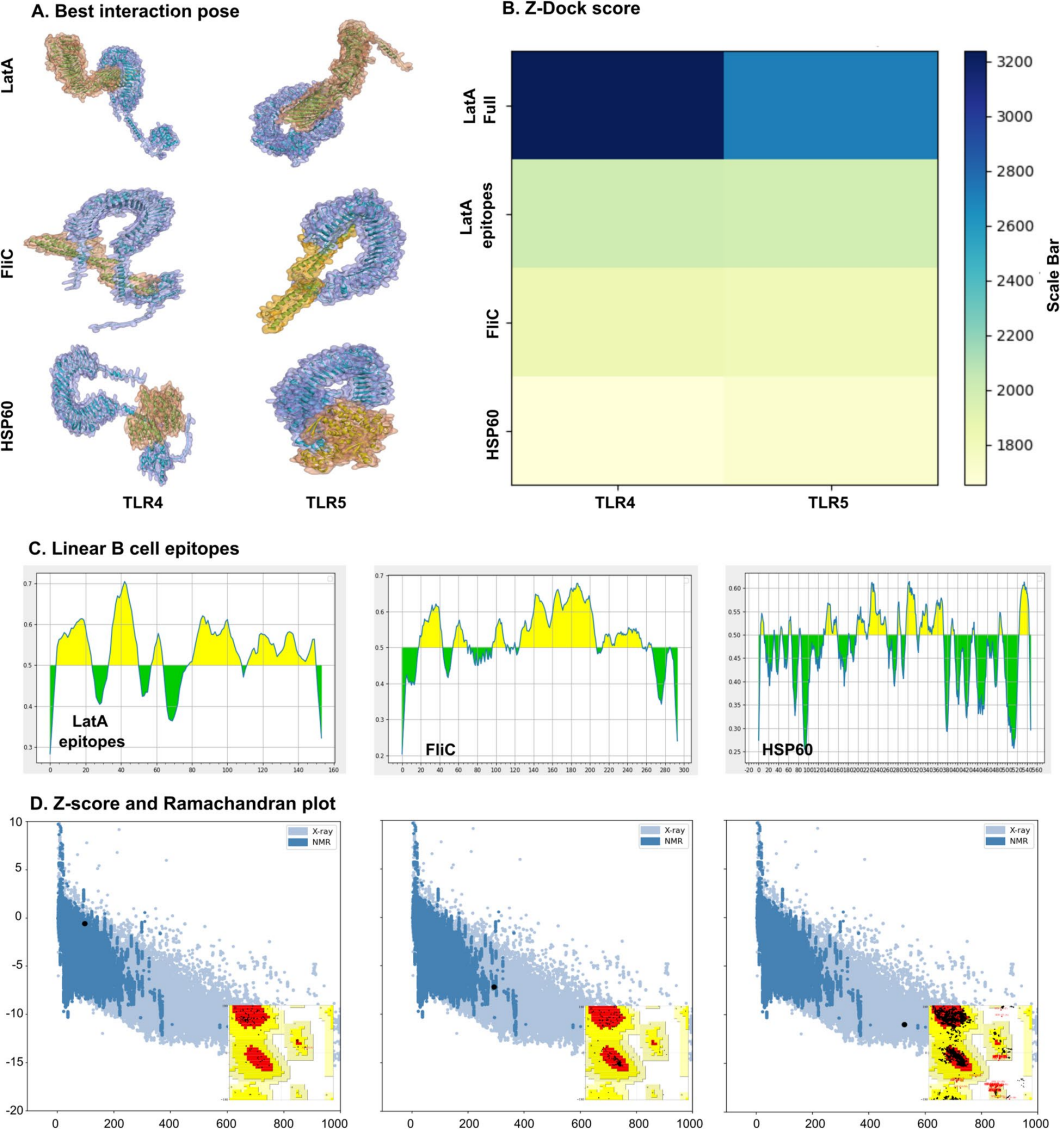
**Molecular docking, epitope mapping, and structural validation of L. intracellularis candidate proteins.** **A** Best interaction poses for LatA, FliC, and HSP60 proteins in complex with TLR4 and TLR5, visualized using docking simulations. **B** Z-Dock scores of each protein-receptor interaction are presented as a heatmap, with higher scores indicating more favorable docking (LatA full, LatA epitopes, FliC, and HSP60 against TLR4 and TLR5). **C** Prediction of linear B cell epitopes for LatA epitopes, FliC, and HSP60 using B cell epitope prediction tools; yellow regions represent predicted epitope regions. **D** Structural validation by ProSA-web Z-score analysis and Ramachandran plots for LatA epitopes, FliC, and HSP60. The calculated Z-scores for LatA epitopes, FliC, and HSP60 models were −0.59, −7.19, and −11.01, respectively, indicating the degree of structure quality. Ramachandran plot shows the structural validation of the antigens.

The gene sequences of *L. intracellularis* antigens were obtained from the NCBI database. The molecular simulations of LatA, FliC, and HSP60 with TLR-4 and TLR-5 were analyzed using the ZDOCK server, and the docking models revealed that the proteins could interact with both TLR-4 and TLR-5. In particular, FliC exhibited a deep insertion into the concave region of both receptors, consistent with its known recognition by TLR5. LatA showed a tighter interaction with TLR-4, while HSP60 formed broader contacts with both receptors (Figure 1A). To assess the binding potential of bacterial ligands with TLRs, the top ZDOCK score was analyzed for each ligand-receptor pair (Figure 1B). These results support the hypothesis that structural diversity among bacterial ligands influences TLR recognition and could drive differential immune signaling.

The overall structural integrity of the targeted antigens, LatA epitopes, FliC, and HSP60, was predicted using bioinformatics tools. An IEDB analysis was performed to predict immunogenic B-cell epitopes of the antigens. The yellow-highlighted amino acids with a score higher than 0.5 (Figure 1C) describe the potential to elicit a humoral response. Ramachandran plots were used to assess model accuracy, revealing that the LatA epitopes and FliC have no residues in disallowed regions. In contrast, only 0.1% of residues were found in disallowed regions for HSP60. The number of residues in the favored areas were 91.0%, 94.7%, and 93.9% for LatA epitopes, FliC, and HSP60, respectively. Furthermore, ProSa-web was used to assess the local structural quality of the predicted structures, indicating good structural quality with Z-scores of −0.59, −7.19, and −11.01 for the LatA epitopes, FliC, and HSP60, respectively (Figure 1D). The overall protective antigen prediction score was calculated using Vaxijen v2.0, yielding probable antigenicity values of 0.8135, 0.6035, and 0.4345 for LatA epitopes, FliC, and HSP60, respectively. Protein-Sol was used to predict the scaled solubility, showing LatA (0.820), FliC (0.449), and HSP60 (0.637). Immunogenic epitopes of the LatA, FliC, and HSP60 genes were codon-optimized and commercially synthesized for cloning into the dual bacterial-host expression plasmids, pJHL270 and pJHL305.

### Designing the plasmids and vaccine constructs

**Figure 2:**
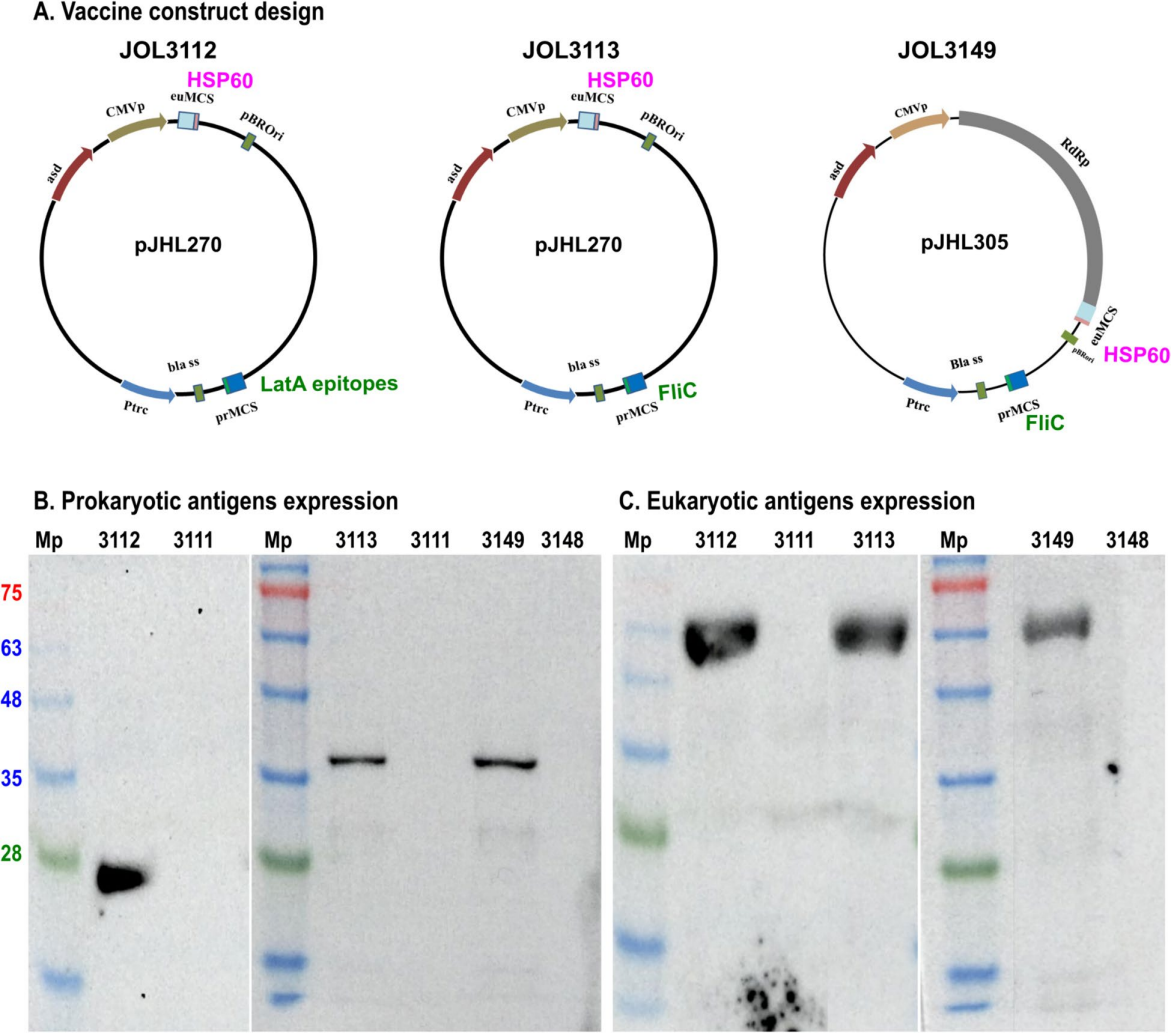
**Vaccine construct design and antigen expression analysis.** **A** Schematic diagrams of vaccine plasmids used in the study as JOL3112 (pJHL270—Pro LatA epitopes + Eu HSP60), JOL3113 (pJHL270—Pro FliC + Eu HSP60), and JOL3149 (pJHL305—Pro FliC + Eu HSP60). **B** Western blot analysis of prokaryotic antigen expression in bacterial cell lysates of *Salmonella* vaccine strains JOL3112 (LatA epitopes), JOL3113 (FliC), JOL3149 (FliC), and their vector controls. The immunoreactive bands observed at approximately 24 kDa and 36 kDa correspond to the expected sizes of LatA epitopes and FliC, respectively. **C** Western blot detection of eukaryotic antigen (HSP60) expression in RAW 264.7 cells infected with JOL3112, JOL3113, and JOL3149. The HSP60 expression (expected size: 60 kDa) was confirmed in the respective lanes. Moreover, no bands were present in the vector controls. Mp: protein marker.

Two dual-expression plasmids constructed by our lab, pJHL270 and pJHL305, were selected for the eukaryotic and prokaryotic expression of *L. intracellularis* recombinant immunogens. Both plasmids contain cytomegalovirus (CMV) and Ptrc promoters for eukaryotic and prokaryotic expression, respectively. The β-lactamase signal sequence (bla SS) was incorporated to facilitate the translocation of recombinant antigens into the periplasm for prokaryotic expression. To ensure constitutive expression in the transformed cells under Darwinian selection pressure, the *asd* gene was incorporated into both plasmids (Figure 2A).

Gene fragments encoding *latA* epitopes and *fliC* were cloned into suitable prokaryotic MCS sites of the plasmids, directing transcription and translation in *Salmonella* via the Ptrc promoter. Similarly, *hsp60* was commercially synthesized and cloned into the eukaryotic MCS under the control of the CMV promoter. The resulting clones were transformed into *E. coli* strain lacking *asd* and electroporated into JOL3090 (ST: *Δlon ΔpagL Δasd*), yielding the vaccine strains JOL3112, JOL3113, and JOL3149, respectively. The respective empty vectors pJHL270 and pJHL305 were electroporated to obtain vector control strains, JOL3111 and JOL3148.

### Validation of antigen expression by western blot

The *L. intracellularis* antigens cloned into the prokaryotic and eukaryotic MCS of pJHL270 and pJHL305 were confirmed by double digestion with restriction enzymes. Furthermore, the prokaryotic expressions of LatA epitopes and FliC were confirmed by western blotting using bacterial cell lysates from the respective vaccine constructs, JOL3112, JOL3113, and JOL3149. The dark immunoreactive bands at 24 kDa and 36 kDa were detected for LatA epitopes and FliC in the vaccine constructs (Figure 2B). Moreover, the expression of HSP60 was validated in RAW 264.7 cells infected with *Salmonella* vaccine strains, as evidenced by a western blot showing a band at 60 kDa (Figure 2C).

### In vitro and in vivo characterization of *Salmonella* delivery strain

**Figure 3:**
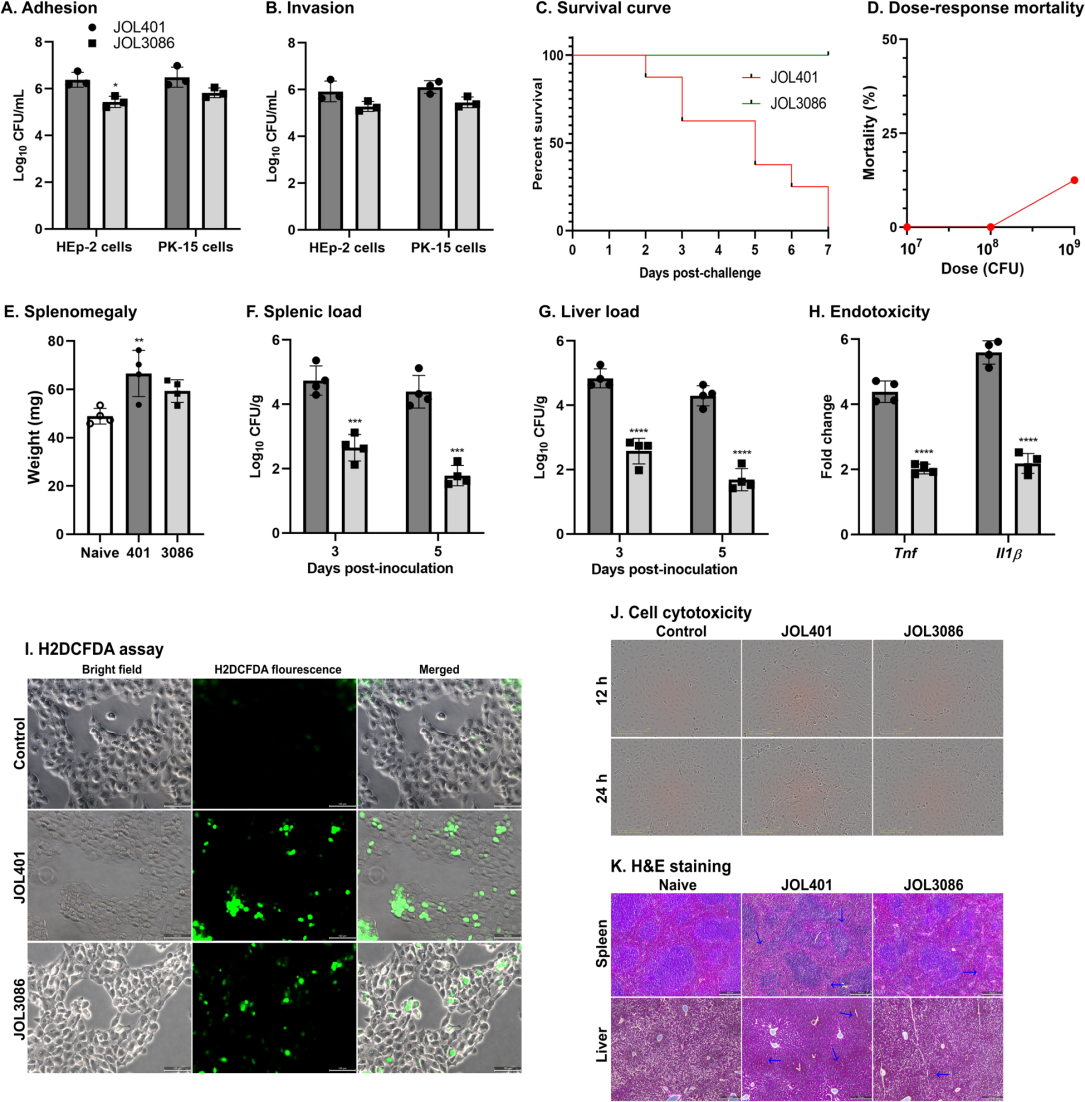
**In vitro and in vivo characterization of** ***Salmonella*** **delivery strain.** **A** Adhesion and **B** Invasion abilities of the wild-type *Salmonella* strain (JOL401) and the delivery strain (JOL3086) were evaluated in HEp-2 (human epithelial) and PK-15 (porcine kidney) cells. **C** Survival curves of C57BL/6 orally inoculated with wild-type (JOL401) or mutant (JOL3086). **D** Dose–response mortality with increasing doses (10^7^, 10^8^, and 10^9^ CFU) of JOL3086, with mortality recorded over 14 days. **E** Spleen weights were measured as an indicator of splenomegaly in naive, JOL401, and JOL3086-inoculated mice at day 3 postinoculation. **F** Bacterial loads in the spleen at days 3 and 5 postinoculation. **G** Bacterial load in the liver at days 3 and 5 postinoculation. **H** Endotoxicity assessment via proinflammatory cytokines TNF-α and IL-1β expression in spleen tissues at day 3 postinoculation. **I** H_2_DCFDA assay showing intracellular ROS generation. Bright-field, fluorescence, and merged images of PK-15 cells infected with JOL401 and JOL3086 or control. Increased green fluorescence indicates higher ROS levels. **J** *Salmonella*-induced cytotoxicity in MARC-145 cells was evaluated by propidium iodide staining and monitored in real time using the IncuCyte live-cell imaging system following infection with JOL401 or JOL3086. Visual assessment revealed a higher number of red-fluorescent objects in JOL401-infected cells. **K** Representative hematoxylin and eosin (H&E) stained sections of spleen and liver at day 3 postinoculation; arrows indicate pathological changes. The data are presented as mean ± SD. Statistical significance: *p < 0.05, **p < 0.01, ***p < 0.001, and ****p < 0.0001.

To assess the interaction between the *Salmonella* delivery strain and host cells, adhesion and invasion assays were performed using the HEp-2 and PK-15 cell lines. As shown in Figure 3A and B, both JOL401 and JOL3086 efficiently adhered to and invaded epithelial cells. In HEp-2 cells, JOL3086 exhibited reduced adhesion compared with the wild-type strain JOL401, while no significant difference was observed in the host PK-15 cells. Similarly, no significant differences in invasion efficiency were observed in either cell line. These results indicate that the *Salmonella* delivery strain retained its ability to adhere to and invade epithelial cells, supporting its potential as a viable delivery vehicle for *L. intracellularis* antigens.

The safety, bacterial burden, and endotoxicity of the vaccine delivery strain (JOL3086) were compared with those of the wild-type (JOL401). We observed clinical symptoms, including squinted eyes, reduced movement, and a hunched posture, in mice inoculated with JOL401. The mice in the JOL401 group showed 100% mortality within 7 days of the inoculation. In contrast, all mice in JOL3086 survived, demonstrating the safety of the mutant strain (Figure 3C).

Furthermore, we tested the pathogenicity of the delivery strain via dose–response mortality. Mice were orally inoculated with the mutant *Salmonella* strain (JOL3086) at different doses, 10^7^, 10^8^, and 10^9^ CFU. No mortality was observed at 10^7^ and 10^8^ CFU, while 1/8 (12.5%) of the mice died at the dose of 10^9^ CFU (Figure 3D). Since mortality did not exceed 50% even at the highest tested dose (10^9^), the LD_50_ of the mutant strain is estimated to be > 10^9^ CFU. These results indicate that the mutant is markedly attenuated compared with wild-type *Salmonella*.

On day 3 postinoculation, there was no significant difference in splenomegaly between JOL3086 and naive control (Figure 3E). We observed a significant reduction in the colonization of JOL3086 in spleen and liver at days 3 and 5 postinoculation, and it was readily cleared by day 7 (Figures 3F and G). Moreover, the endotoxicity of the delivery strain was assessed by measuring proinflammatory cytokine levels in spleens collected on day 3 postinoculation. Compared with the JOL401 group, mice inoculated with JOL3086 had significantly lower levels of *Tnf* and *Il1b* mRNA (Figure 3H).

To evaluate oxidative stress induced by the *Salmonella* vaccine delivery strain, PK-15 cells were stained with the ROS-sensitive dye H_2_DCFDA. The cells infected with JOL401 and JOL3086 exhibited pronounced green fluorescence, indicating elevated ROS generation compared with the mock control (Figure 3I). The increased fluorescence intensity suggests enhanced oxidative activity associated with bacterial internalization and immune stimulation.

Intracellular cytotoxicity caused by JOL401 and JOL3086 was examined through propidium iodide staining. MARC-145 cells were monitored in real-time with the IncuCyte live imaging system (Figure 3J). At 12 h and 24 h, visual assessment revealed a higher number of red-fluorescent objects in cells infected with the ST WT JOL401 strain.

Furthermore, histopathological examination by H&E staining revealed prominent tissue distortion in the spleen of WT *Salmonella*-infected mice. Immune cell infiltration was noted in the liver infected with WT *Salmonella* as a sign of inflammation (Figure 3K). Overall, these findings suggest that modifying the lipid A of the ST strain resulted in a profound reduction in endotoxicity and improved safety.

### Post-immunization immunoglobulin responses

**Figure 4:**
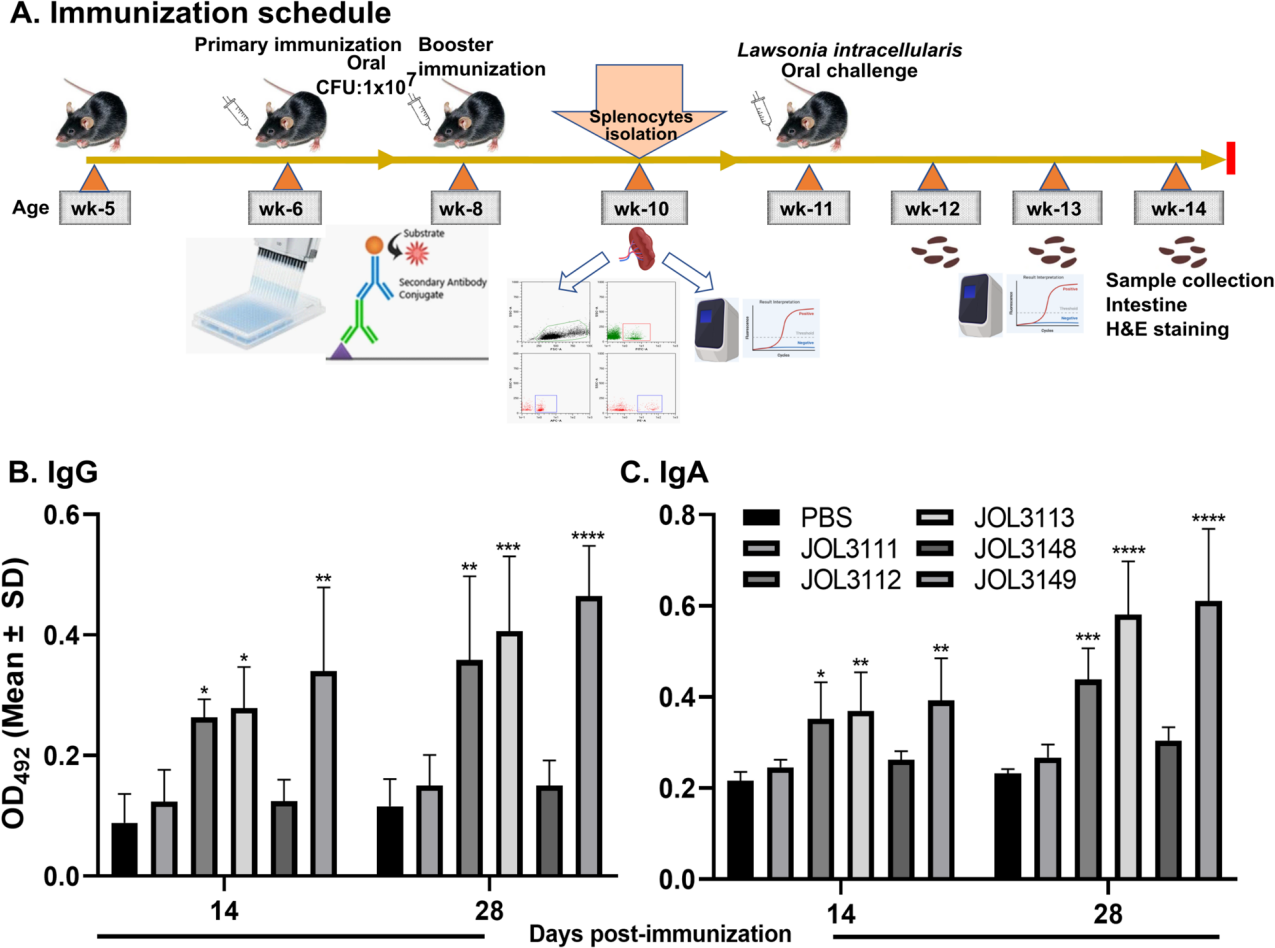
**Immunization scheme and antibody response in mice immunized with *Salmonella*-based L. intracellularis vaccine candidates**. **A** Immunization schedule for C57BL/6 female mice. **B** Systemic IgG responses measured in sera by indirect ELISA at 14 and 28 days after immunization. **C** Mucosal IgA responses were measured in vaginal wash samples by indirect ELISA at 14 and 28 days after vaccination. The data are presented as mean ± SD (*n* = 4 per group), with significant differences indicated as *p < 0.05, **p < 0.01, ***p < 0.001, and ****p < 0.0001 compared with the PBS control.

The mice were immunized as shown in Figure 4A, and serum and vaginal wash samples were collected from vaccinated and control mice at 2 weeks after the primary and booster immunizations. IgG and IgA antibody responses in the immunized sera and vaginal wash samples were evaluated by indirect ELISA using *L. intracellularis*-specific proteins. The vaccine strains JOL3112, JOL3113, and JOL3149 induced significantly higher IgG levels than the PBS control at both 14- and 28-day postimmunization time points, indicating a strong systemic immune response (Figure 4B).

Aside from monitoring IgG antibody levels, mucosal IgA levels in vaginal wash samples were also measured. At both 14- and 28-day postimmunization, IgA antibody levels were significantly elevated in the JOL3112, JOL3113, and JOL3149 groups compared with the PBS control, suggesting a significant higher mucosal immune response (Figure 4C).

Overall, the JOL3149 vaccine strain induced the highest levels of both systemic IgG and mucosal IgA immune responses, supporting its potential as an effective vaccine candidate.

### Cell-mediated immune responses

**Figure 5:**
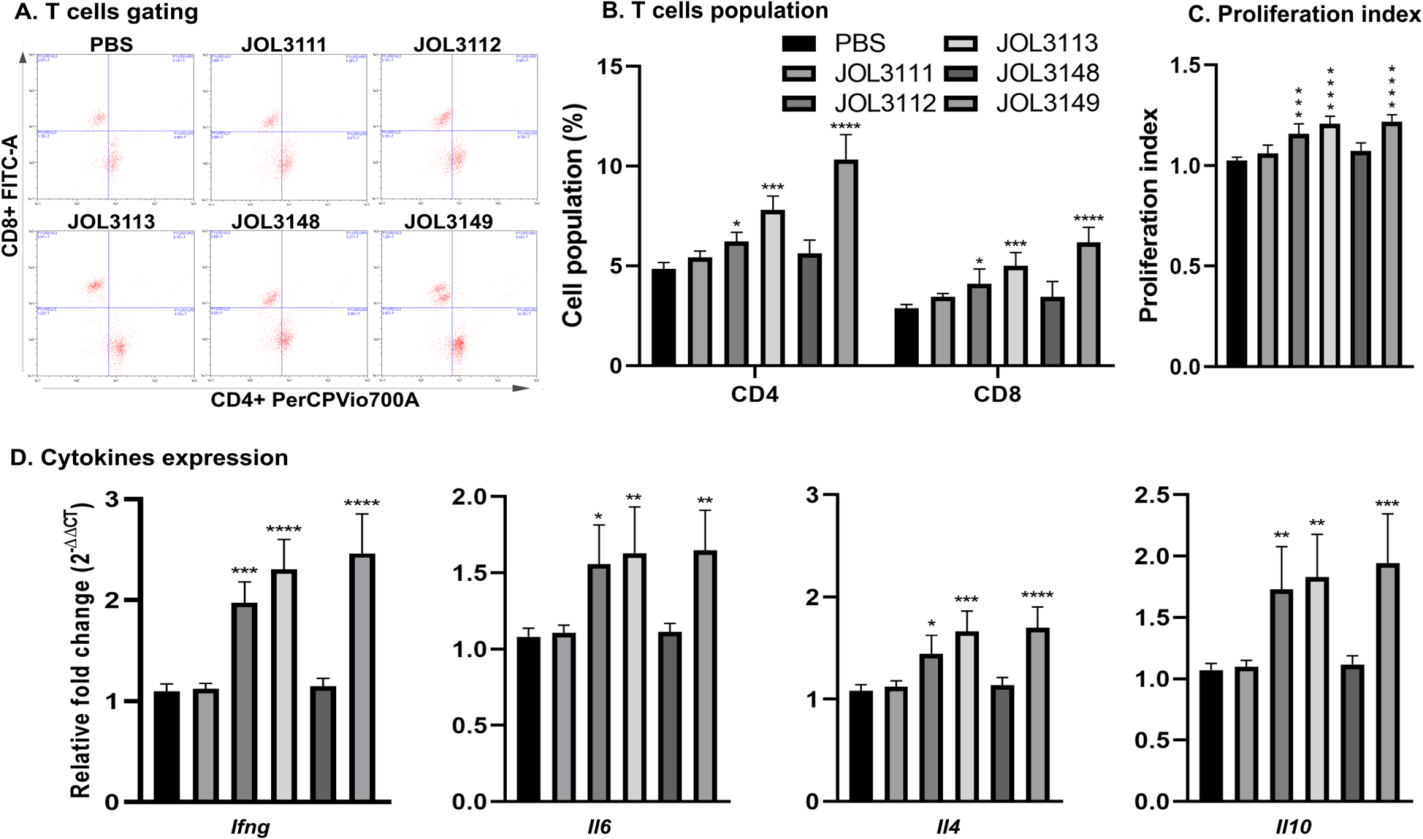
**Analysis of T cell responses following immunization with recombinant *Salmonella* vaccine strains**. **A** Representative fluorescence-activated cell sorting (FACS) gating plots show the proportions of CD4^+^ and CD8^+^ T cells among splenocytes from PBS control and vaccinated groups. **B** Quantification of CD4^+^ and CD8^+^ T cell populations among splenocytes from each experimental group. Vaccine groups JOL3112, JOL3113, and JOL3149 showed a statistically significant increase in CD4^+^ and CD8^+^ T cell populations compared with the PBS control group, indicating higher cellular immune responses. **C** Splenocyte proliferation index, demonstrating enhanced cellular proliferation in vaccine groups. **D** Quantitative real-time PCR (qRT-PCR) analysis of cytokine gene expression (*Ifng*, *Il6*, *Il4*, *Il10*) in splenocytes from immunized mice. JOL3112, JOL3113, and JOL3149 vaccine strains significantly upregulated cytokine expression, indicating induction of Th1/Th2 cytokine responses against *L. intracellularis*. Data presented as mean ± SD (*n* = 4 per group). Statistical significance: *p < 0.05, **p < 0.01, ***p < 0.001, ****p < 0.0001.

Eliciting cellular immunity has also been regarded as a noteworthy factor in identifying a promising vaccine candidate. Splenocytes were analyzed by flow cytometry to determine T cell subset populations in immunized mice at 14 days after booster immunization. The results revealed a significant increase in the CD4^+^ and CD8^+^ T-cell populations in the mice immunized with recombinant *Salmonella* strains. Among the experimental groups, JOL3112, JOL3113, and JOL3149 increased the CD4^+^ population, with a statistically significant difference compared with the PBS control group. Similarly, CD8^+^ T cell populations were significantly increased in JOL3112, JOL3113, and JOL3149 groups. Representative images of the T-cell gating are shown in Figure 5A, and the data indicate the ability of our vaccine strains, particularly JOL3149, to elicit the most significant cellular immune response (Figure 5B).

The MTT assay indicated a marked increase in proliferation indices among the vaccinated groups. In particular, JOL3113 and JOL3149 exhibited a significantly higher proliferation index (*p* < 0.0001) than the PBS control (Figure 5C). The findings indicate that immunization with recombinant *Salmonella* strains effectively induces strong cellular immune responses, as evidenced by significantly enhanced T cell activation and proliferation.

### Cytokines response

Cellular immunity was further evaluated in immunized mice by assessing cytokine responses. The induction of immunomodulatory cytokines was measured at the mRNA levels using qRT-PCR. The analysis revealed that JOL3112, JOL3113, and JOL3149 significantly upregulated *Ifng*, *Il6*, *Il4*, and *Il10* compared with PBS control. These results demonstrate that our vaccine strains elicited a strong immune response, favoring a Th1-Th2-balanced response against *L. intracellularis* infection (Figure 5D).

### Protection against *L. intracellularis* challenge

**Figure 6:**
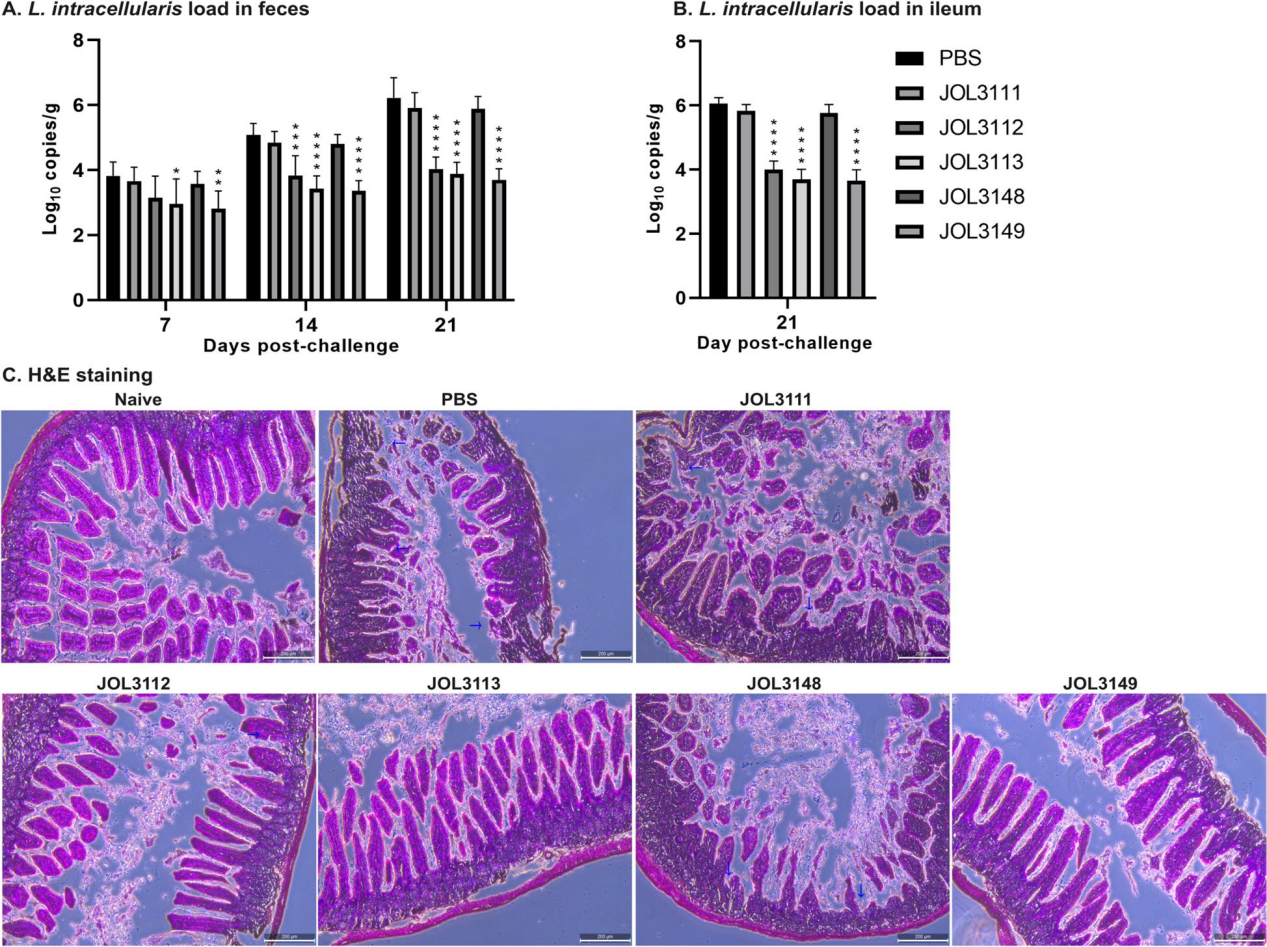
***Quantification of Ls.* intracellularis load and histopathological changes in challenged mice**. **A** *Lawsonia intracellularis* load in feces collected at days 7, 14, and 21 postchallenge was quantified by qRT-PCR targeting the *aspA* gene. Vaccine groups demonstrated significantly reduced bacterial shedding compared with the PBS control at multiple time points. **B** *Lawsonia intracellularis* load in ileal tissues at day 21 postchallenge was measured, and all vaccinated groups exhibited a reduced bacterial burden relative to the PBS control. **C** Representative hematoxylin and eosin (H&E) stained intestine sections harvested at 21 days postchallenge, illustrating intestinal architecture. The immunized groups showed relatively preserved villous structures and substantially less pathology. Blue arrows indicate areas of damage. Scale bar: 200 µm. The data are presented as mean ± SD (*n* = 5 per group), with significant differences indicated as *p < 0.05, **p < 0.01, ***p < 0.001, and ****p < 0.0001 compared with the PBS control.

The vaccine strains were designed to achieve protection against *L. intracellularis.* In the third week after booster immunization, mice were orally challenged with 5 × 10^7^ *L. intracellularis.* Fecal bacterial shedding of *L. intracellularis* was monitored at 7, 14, and 21 days postchallenge. As shown in Figure 6A, the immunized groups demonstrated a significant reduction in the fecal bacterial load, compared with the PBS-challenged group. In particular, on day 21 postchallenge, the JOL3149 vaccine strain showed a 2.52 log_10_ reduction in fecal shedding of *L. intracellularis*. Consistent with these findings, ileal tissues collected at day 21 postchallenge showed a significantly lower bacterial load (2.40 log_10_ reduction) in JOL3149-vaccinated mice compared with PBS control (Figure 6B). Histopathological examination further revealed that PBS-challenged mice developed severe lesions with distorted and disturbed villi structures. In contrast, immunized groups, especially JOL3149, showed only mild lesions (Figure 6C).

### Comparative protection analysis with a commercial vaccine

At day 21 postchallenge, mice immunized with JOL3149 or the live commercial vaccine (CV) exhibited a significant reduction in fecal bacterial shedding compared with the PBS control (Additional file 2A). Consistent with these findings, histopathological examination of intestinal tissues revealed mild lesions in both JOL3149- and CV-immunized groups. In contrast, pronounced pathological changes in intestinal structures were observed in the PBS group (Additional file 2B).

## Discussion

The present study reports the successful engineering and immunological evaluation of a genetically attenuated *Salmonella* Typhimurium (ST) strain expressing *L. intracellularis* antigens as potential live vaccine candidates. Through a systematic approach integrating molecular engineering, immunoinformatics, and in vivo immunological assays, we demonstrated that the engineered vaccine strains elicited humoral and cell-mediated immune responses while maintaining a wide safety margin. The results collectively highlight the feasibility of using a lipid A-modified *Salmonella* delivery platform for mucosal vaccination against *L. intracellularis* infection.

The selection of *L. intracellularis* antigens, LatA, FliC, and HSP60, was guided by their potential roles in host–pathogen interaction and immunogenic properties. In silico docking analysis revealed specific interactions between these antigens and Toll-like receptors TLR4 and TLR5, suggesting their ability to activate innate immune signaling pathways crucial for early defense against intracellular bacteria. The strong binding of LatA to TLR4 observed in the docking analysis implies its potential to contribute to macrophage activation and cytokine production. Thus, we selected immunoprotective epitopes of a surface autotransporter protein, LatA, which is functionally linked to the type V secretion system. A flagellar protein (FliC), involved in host invasion, was included in this study owing to its ability to activate TLR5-mediated immunity and promote adaptive responses [31]. In addition, HSP60 was selected for its established role in protective immunity across pathogens [18, 32, 59]. HSP60 is a conserved chaperone protein, indicating a broader immunostimulatory role and potential adjuvant-like activity (Figure 1A). It shows that the antigen can interact favorably with host TLR4 and TLR5 receptors [29], as reflected by the Z-Dock score (Figure 1B). Linear B-cell epitope mapping further identifies immunogenic regions across the three antigens, supporting their suitability as vaccine components (Figure 1C). Structural validation using ProSA-web Z-scores and Ramachandran plots further confirms the quality of the modeled proteins (Figure 1D).

Traditional subunit vaccines deliver antigens extracellularly, whereas mRNA vaccines elicit immune responses via intracellular antigen presentation [60]. We engineered dual-promoter systems incorporating Ptrc and CMV promoters to integrate both strategies. Our plasmids, pJHL270, and pJHL305, enhance antigen presentation via both exogenous and endogenous pathways. They enable MHC class I and MHC class II-mediated presentation, activating CD8 cytotoxic and CD4 helper T cells. In this way, they induce cellular and humoral immune responses. *Salmonella* carrying Ptrc-driven plasmids mediate exogenous antigen delivery, while host cell invasion triggers CMV-driven endogenous expression. Furthermore, pJHL305 employs an RdRp amplification system to increase mRNA levels, thereby enhancing CD8 T-cell activation [49, 61]. Codon optimization and expression validation in both prokaryotic and eukaryotic systems (Figure 2A) ensured that the recombinant proteins were expressed properly and were immunologically accessible. Moreover, western blot analysis confirmed the expected molecular weights of LatA epitopes (24 kDa), FliC (36 kDa), and HSP60 (60 kDa), verifying successful antigen expression in both bacterial and mammalian cells (Figures 2B and C). These results demonstrate that the dual-expression plasmid systems (pJHL270 and pJHL305) enable efficient and simultaneous expression of both antigens.

While the expression vector system is fundamental, the proper delivery of the antigen-encoding plasmid remains a key determinant for optimal gene expression and immunogenicity [62]. We engineered a novel *Salmonella* Typhimurium (ST) delivery system by deleting the *lon* and *pagL* genes using λ-red recombinant technology. In line with previous reports on attenuated *Salmonella* strains, the lipid A-modified mutant JOL3086 (Δ*lon* Δ*pagL)* showed a safer, less virulent profile than the ST wild-type strain (JOL401) [45, 63, 64]. JOL3086 retained efficient adhesion and invasion of epithelial and kidney cells (Figure 3A, B). Still, it showed reduced virulence in vivo, as evidenced by increased survival rates, lower mortality, reduced splenomegaly, and diminished bacterial loads in both the spleen and the liver (Figure 3C–G). JOL3086 also exhibited significantly reduced levels of proinflammatory cytokine responses, confirming its lower endotoxicity (Figure 3H). Furthermore, JOL3086 induced comparable intracellular ROS (Figure 3I), lower cytotoxic response (Figure 3J), and minimal pathological changes in host tissues (Figure 3K). These results collectively demonstrate that rational attenuation of *Salmonella* minimizes host toxicity while maintaining immunogenicity, supporting JOL3086 as a promising, safe vaccine delivery platform.

Oral immunization with the recombinant *Salmonella* vaccine strains (Figure 4A) induced strong systemic and mucosal immune responses, characterized by significant elevations of serum IgG and mucosal IgA levels. Among the vaccine constructs, JOL3149 elicited the highest IgG and IgA responses (Figure 4B, C), suggesting superior immunogenicity. The elevated IgA levels in vaginal washes indicate efficient mucosal priming, consistent with the established ability of *Salmonella*-based vaccines to induce mucosal immunity through stimulation of gut-associated lymphoid tissue. It shows that antigens of *L. intracellularis* from *Salmonella* and host cells interact with immune cells to elicit an immune response, thereby stimulating protective immunity [65–68].

Beyond humoral immunity, effective protection against intracellular bacteria depends on cellular immune mechanisms. In pigs with prior *L. intracellularis* infection, reinfection elicited a significant increase in IFN-γ production, indicating the induction of T cell memory responses against *L. intracellularis* [69]. Likewise, studies in IFNγ-deficient mice demonstrated markedly higher susceptibility to *L. intracellularis* challenge, with increased mortality, underscoring the essential role of T cell-mediated immunity in protective mechanisms [70]. Consistent with these findings, our vaccine strains, JOL3112, JOL3113, and JOL3149, induced statistically significant differences in CD4 and CD8 T-cell populations (Figures 5A, B), accompanied by high proliferation indices in the MTT assay, suggesting efficient T cell expansion (Figure 5C). Furthermore, immunized mice exhibited significantly elevated levels of *Ifng*, *Il6*, *Il4*, and *Il10* (Figure 5D), reflecting activation of both Th1 and Th2 responses. Considering that *L. intracellularis* infection leads to immunosuppression, an effective vaccine should elicit coordinated mucosal and cellular immunity to confer adequate protection [71]. Among the tested strains, JOL3149 induced the strongest cellular immune responses, supporting its potential as a promising vaccine candidate against *L. intracellularis* infection.

The ultimate measure of vaccine effectiveness lies in its ability to confer protection against pathogen exposure. The significant reduction in bacterial shedding in feces and ileal tissues following challenge with *L. intracellularis* indicates that the immune response elicited by vaccination reduced bacterial replication and transmission (Figure 6A, B). On day 21 postchallenge, the JOL3149 strain reduced bacterial shedding by 2.52 log_10_ in feces and 2.40 log_10_ in the ileum, aligning with previous findings that effective vaccination markedly decreases pathogen shedding and tissue invasion [18, 72]. Correspondingly, histopathological analyses revealed that vaccination not only reduced pathogen burden but also mitigated intestinal lesion severity (Figure 6C). To address comparative concerns regarding the efficacy of existing commercial vaccines, we performed an additional protection study using the commercial live *L. intracellularis* vaccine, Enterisol® Ileitis. In the murine challenge model, the lead candidate JOL3149 and CV showed significant reductions in fecal bacterial shedding and histopathological lesion severity compared with PBS control (Additional files 2A and B), further supporting the protective potential of the *Salmonella*-mediated dual-expression platform.

**Figure 7:**
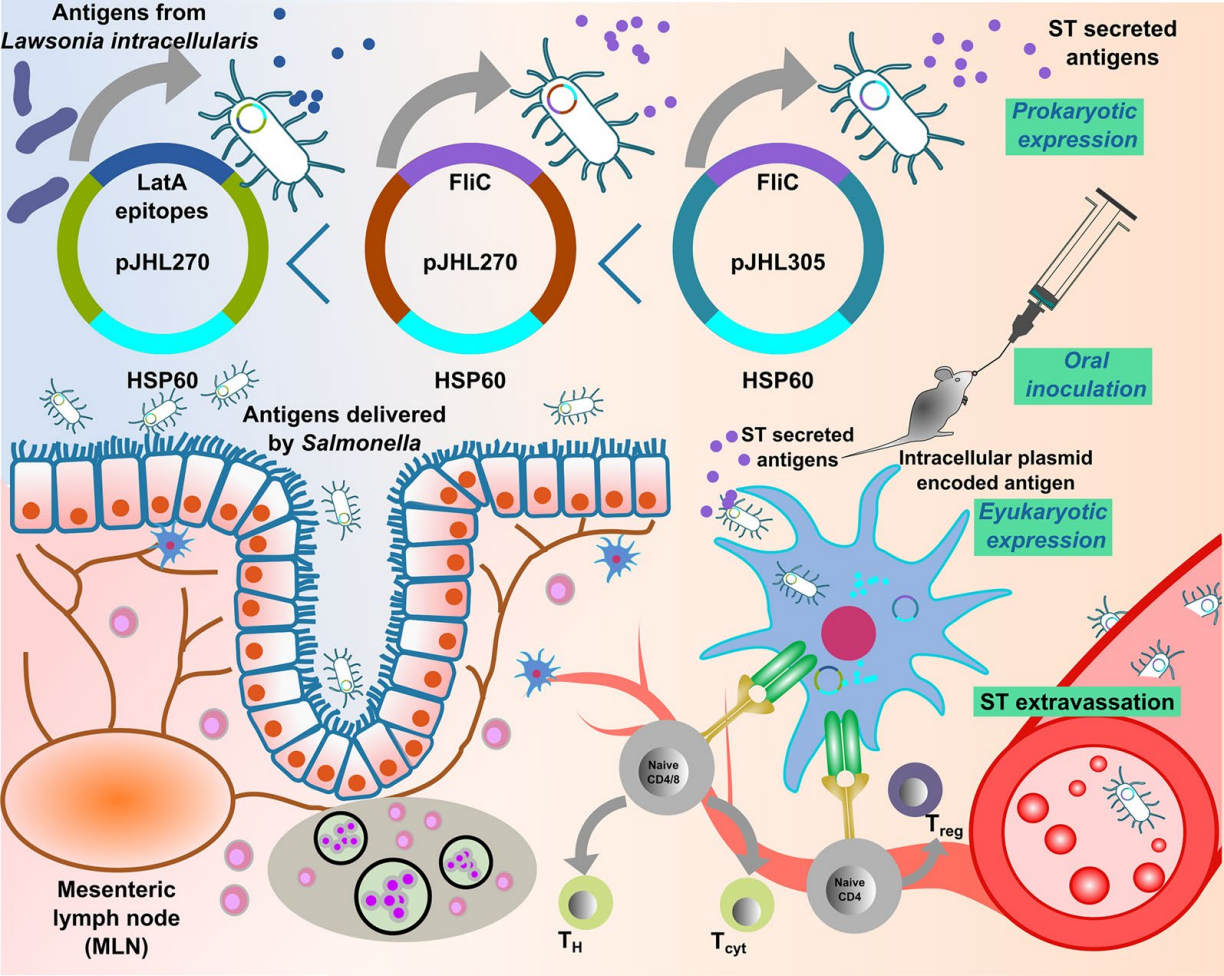
**Strategic mechanism of the *Salmonella-based antigen* delivery system for vaccination against** ***Lawsonia*** **intracellularis**. The figure depicts attenuated *Salmonella Typhimurium* strains harboring plasmids (pJHL270 or pJHL305) engineered to express immunoprotective antigens: LatA epitopes, FliC, and HSP60 from *L. intracellularis*. *Salmonella* vectors deliver antigens via dual mechanisms: prokaryotic secretion of LatA epitopes and FliC, and eukaryotic intracellular expression of HSP60 following oral inoculation. The diagram shows antigen trafficking through intestinal epithelial cells, uptake by antigen-presenting cells, migration to the mesenteric lymph node (MLN), and subsequent activation of helper (TH), cytotoxic (TCyt), and regulatory (Treg) T-cell populations. The inclusion of the RdRp system in pJHL305 enhances cytoplasmic antigen expression, boosting CD8 ^+^ T-cell responses. This workflow highlights the interplay between mucosal and systemic immunity, supporting effective protection against *L. intracellularis.*

These findings emphasize the role of vaccine-induced protective immunity in preventing both clinical and subclinical manifestations of *L. intracellularis* infection. In particular, the vaccine strain JOL3149, which expresses *L. intracellularis* antigens (pro-FliC + Eu-HSP60) via the pJHL305 vector, exhibited superior efficacy in controlling the infection in a murine model. This enhanced performance may be attributed to RdRp activity from the pJHL305 plasmid, which markedly increases cytoplasmic mRNA synthesis and, consequently, improves eukaryotic expression [73]. Furthermore, the use of a bacterial backbone enables rapid, cost-effective vaccine production with enhanced thermal stability. Collectively, the *Salmonella*-mediated vaccine platform, depicted schematically in Figure 7, effectively delivers *L. intracellularis* antigens through complementary prokaryotic and eukaryotic expression pathways. This strategic design promotes mucosal and systemic immunity, providing a promising foundation for future vaccine development against proliferative enteropathy.

Although *L. intracellularis* is mainly a porcine pathogen, the murine model used in this study provides a biologically relevant system for initial evaluation of vaccine immunogenicity and protection [33, 74]. While mice may not develop the full pathological features of porcine proliferative enteropathy, previous studies have demonstrated that *L. intracellularis* antigens can elicit humoral and cellular immune responses in mice that correlate with protective potential in the natural host [18, 33, 74]. Therefore, the mouse model serves as a proof-of-concept platform to evaluate the safety, immunogenicity, and delivery efficiency of the low-endotoxic *Salmonella* vector prior to validation in pigs, which remains the definitive target species.

## Conclusions

This study establishes a rationally designed *Salmonella*-based mucosal vaccine platform that combines lipid A modification for safety with dual-expression plasmids for efficient antigen delivery. The integrated immunoinformatic-guided antigen selection and in vivo immunological validation provide a framework for developing vaccines against intracellular pathogens such as *L. intracellularis*. The strong humoral, mucosal, and cellular immune responses, along with decreased fecal and ileal bacterial shedding and tissue colonization, underscore the protective potential of the vaccine constructs, particularly JOL3149.

## Supplementary Information

**Additional file 1**. Confirmation of deletion of the *pagL* and *asd* genes.

**Additional file 2**. Comparative protective efficacy of JOL3149 and commercial live *L. intracellularis* vaccines.

## Data Availability

Data will be made available on reasonable request from the corresponding author.
