# Supplementary material for: A low-endotoxic Salmonella vector with dual bacterial-host promoter expression of Lawsonia intracellularis antigens elicits protective immunity in a murine model
Source: Vet Res. 2026 Mar 21;57:59. doi: 10.1186/s13567-026-01726-w (PMC13126819; doi:10.1186/s13567-026-01726-w)
Supplement: Supplementary file 1 — Additional file 1. Confirmation of deletion of the pagL and asd genes. [file 13567_2026_1726_MOESM1_ESM.docx]

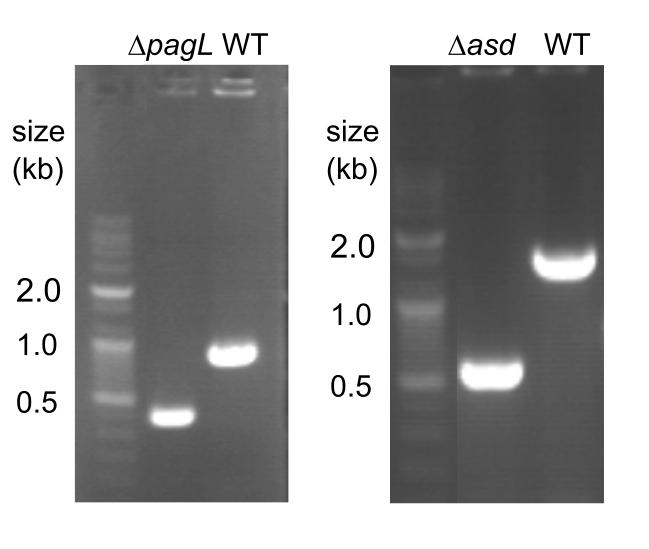


**Additional file 1** **Confirmation of deletion of the *pagL* and *asd* genes.** The parent strain JOL909 was used to delete the *pagL* and *asd* genes via the lambda red recombination technique, and the gene deletions were confirmed by PCR using specific outer primers.
