# Supplementary material for: A low-endotoxic Salmonella vector with dual bacterial-host promoter expression of Lawsonia intracellularis antigens elicits protective immunity in a murine model
Source: Vet Res. 2026 Mar 21;57:59. doi: 10.1186/s13567-026-01726-w (PMC13126819; doi:10.1186/s13567-026-01726-w)
Supplement: Supplementary file 2 — Additional file 2. Comparative protective efficacy of JOL3149 and commercial live L. intracellularis vaccines. [file 13567_2026_1726_MOESM2_ESM.docx]

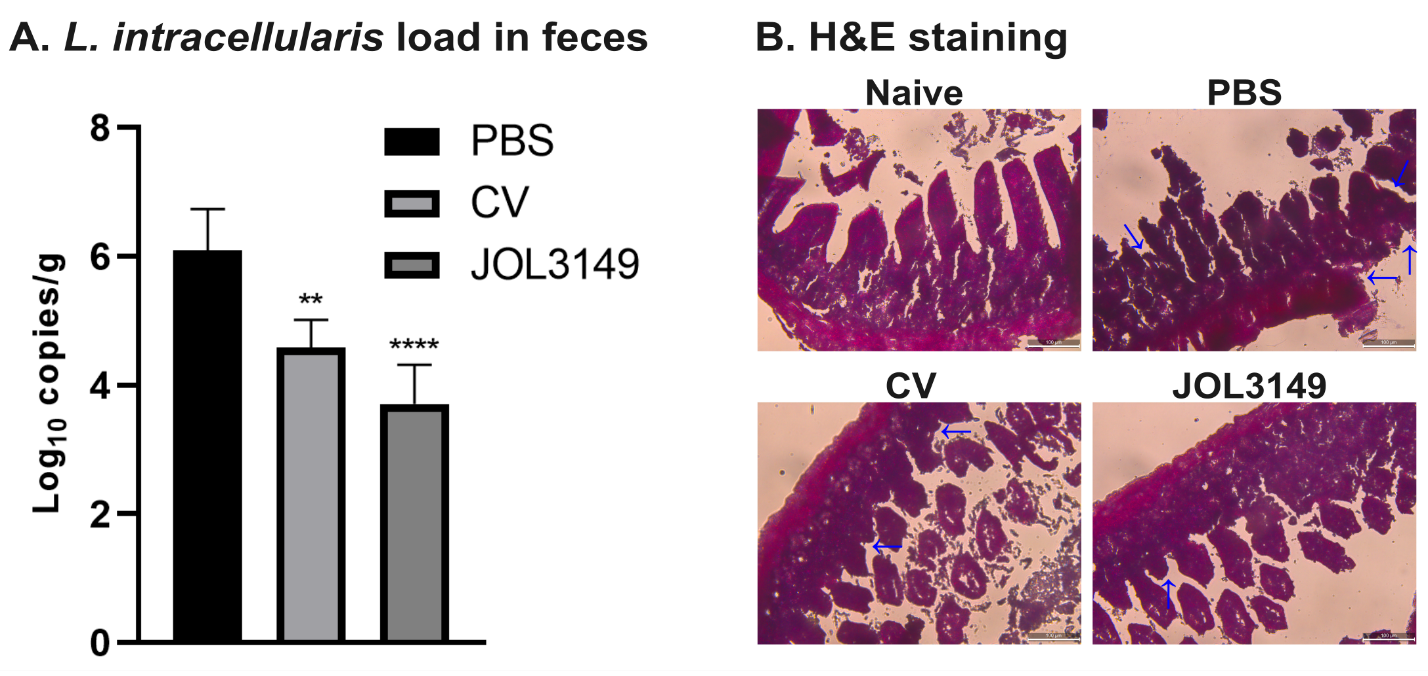


**Additional file 2 Comparative protective efficacy of JOL3149 and commercial live *L. intracellularis* vaccine. A** Fecal bacterial shedding at day 21 post-challenge was quantified by qRT-PCR. Mice immunized with the commercial vaccine (CV) or JOL3149 showed a significant reduction in bacterial shedding compared with the PBS control group. **B** Histopathological lesions in ileal tissues collected at day 21 post-challenge were evaluated following hematoxylin and eosin (H&E) staining. JOL3149 and CV groups exhibited mild lesions and relatively preserved intestinal architecture compared with the PBS group. Scale bar: 100 µm. Data are presented as mean ± SD (n = 5 per group). Statistical significance is indicated as ***p* < 0.01, and *****p* < 0.0001 compared with the PBS control.
